# Relation Between Mathematical Performance, Math Anxiety, and Affective Priming in Children With and Without Developmental Dyscalculia

**DOI:** 10.3389/fpsyg.2018.00263

**Published:** 2018-04-26

**Authors:** Karin Kucian, Isabelle Zuber, Juliane Kohn, Nadine Poltz, Anne Wyschkon, Günter Esser, Michael von Aster

**Affiliations:** ^1^Center for MR-Research, University Children's Hospital, Zurich, Switzerland; ^2^Children's Research Center, University Children's Hospital, Zurich, Switzerland; ^3^Neuroscience Center Zurich, University of Zurich, ETH Zurich, Zurich, Switzerland; ^4^Department of Psychology, University of Potsdam, Potsdam, Germany; ^5^Academy for Psychotherapy and Intervention Research, University of Potsdam, Potsdam, Germany; ^6^Clinic for Child and Adolescent Psychiatry, German Red Cross Hospitals, Berlin, Germany

**Keywords:** developmental dyscalculia, mathematics, affective priming, calculation, arithmetic, anxiety, gender, children

## Abstract

Many children show negative emotions related to mathematics and some even develop mathematics anxiety. The present study focused on the relation between negative emotions and arithmetical performance in children with and without developmental dyscalculia (DD) using an affective priming task. Previous findings suggested that arithmetic performance is influenced if an affective prime precedes the presentation of an arithmetic problem. In children with DD specifically, responses to arithmetic operations are supposed to be facilitated by both negative and mathematics-related primes (=*negative math priming effect*).We investigated mathematical performance, math anxiety, and the domain-general abilities of 172 primary school children (76 with DD and 96 controls). All participants also underwent an affective priming task which consisted of the decision whether a simple arithmetic operation (addition or subtraction) that was preceded by a prime (positive/negative/neutral or mathematics-related) was true or false. Our findings did not reveal a *negative math priming effect* in children with DD. Furthermore, when considering accuracy levels, gender, or math anxiety, the *negative math priming effect* could not be replicated. However, children with DD showed more math anxiety when explicitly assessed by a specific math anxiety interview and showed lower mathematical performance compared to controls. Moreover, math anxiety was equally present in boys and girls, even in the earliest stages of schooling, and interfered negatively with performance. In conclusion, mathematics is often associated with negative emotions that can be manifested in specific math anxiety, particularly in children with DD. Importantly, present findings suggest that in the assessed age group, it is more reliable to judge math anxiety and investigate its effects on mathematical performance explicitly by adequate questionnaires than by an affective math priming task.

## Introduction

Mathematical skills are vital for everyday life and deficits in mathematical performance have negative effects in many domains like education, profession and daily routine. It is commonly known that many children have negative attitudes and emotions toward mathematics (Dowker et al., 2016). In some children the negative emotions toward mathematics may evoke severe anxiety, and as a consequence, these children often avoid mathematical activities.

The literature has proposed different definitions of mathematics anxiety, however, common to most is the observation that dealing with mathematics may evoke a negative emotional response in some people (Suárez-Pellicioni et al., 2016). Mathematics anxiety involves feelings of tension and interferes with mathematical performance (Dowker et al., 2016). Mathematics anxiety is certainly a significant problem that appears to increase with age during childhood. According to the Organization for Economic Co-operation and Development (OECD), 31% of 15-year-old students reported feeling nervous when solving a math problem and as many as 59% indicated that they were worried about math classes (OECD, 2013). Apart from aspects like gender, age and culture affecting mathematics anxiety, research has shown that emotional factors, such as general anxiety or self-esteem play an important role too (Orly Rubinsten and Tannock, 2010; Dowker et al., 2016). Environmental and genetic factors have also been discussed. As associations between mathematics anxiety and achievement have been validated, it is assumed that children with learning disabilities in mathematics show higher levels of mathematics anxiety (Wu et al., 2014). Hence, children suffering from math learning disorders, such as developmental dyscalculia (DD), are of particular interest when investigating these relations. With a prevalence rate of between 3 and 6%, children suffering from DD are clearly not a rare exception (Shalev et al., 2000). DD is a heterogeneous learning impairment affecting numerical and/or arithmetic functioning on the behavioral, psychological and neuronal levels (reviewed by Kucian and von Aster, 2015). Hereditary and environmental factors are presumed to represent possible causes, and children affected from DD report problems with counting, magnitude processing, arithmetic but also more general competences such as working memory or attentional processes. Furthermore, children with DD often suffer from additional psychiatric disorders like depression or anxieties. Anxiety is especially present in the context of mathematics and is associated with stress (reviewed by Dowker et al., 2016).

Despite their relatively high occurrence and significant importance, comparatively little research has been conducted on the interaction between low performance in mathematics and negative emotions. The topic has received increasing attention, yet much remains unexplained and contradictory findings have been reported. Of particular interest is the relation between cognitive abilities and emotional factors and attitudes in mathematical performance (e.g., Dowker et al., 2012). However, the direction of causation is undefined. On the one hand, it is possible that having high mathematics anxiety leads to greater avoidance tendencies in situations that involve mathematics, resulting in less practice and hence lower achievement. On the other hand, it is also plausible that poor mathematical performance promotes mathematics anxiety. Moreover, working memory capacity has been shown to be lower in highly math-anxious subjects (e.g., Mammarella et al., 2015). Interestingly, the authors reported that children with math anxiety are specifically impaired in verbal working memory, whereas children with DD showed specific deficits in visuospatial working memory. Hence, children with math anxiety or with DD may fail in math due to different underlying cognitive impairments in working memory. Although it is unclear to what extent mathematics anxiety causes mathematical difficulties or vice versa, there is conclusive proof that math anxiety interferes with mathematical performance, especially with tasks requiring working memory. The most prominent theory explains this relationship by worrying intrusive thoughts involved in math anxiety that consume attentional working memory resources, such that fewer resources are available for numerical cognition (reviewed by Suárez-Pellicioni et al., 2016). Apart from the general lack of research, particularly little is known about the relationship between mathematics anxiety and performance in young children, as previous studies mostly included older children, adolescents or adults. The understanding of early development, however, is crucial in order to prevent mathematics anxiety and negative emotions in the context of mathematical performance (Wu et al., 2012).

One possible strategy for studying the link between mathematical performance and emotions is by the use of priming tasks. Priming tasks are implicit measures and assess evaluations that are activated automatically after the presentation of a stimulus (Krause et al., 2012). Hence, in priming paradigms, resulting effects are mainly caused by response activation processes (De Houwer et al., 2009). The type of priming which is relevant in the context of mathematics anxiety is affective priming, which, in accordance with standard priming paradigms, consists of a stimulus (prime) and a response to a target. Importantly, in affective priming tasks, the affective relation between prime and target is manipulated since the valence of the prime stimulus is either positive, negative or neutral (Hermans et al., 1994). The idea in affective priming tasks is that “participants are faster at evaluating a target stimulus if a previously presented prime stimulus has the same valence compared to a condition in which a prime stimulus of the opposite valence is shown” (Werner and Rothermund, 2013, p. 119). Hence, if the prime-target pair is of the same valence (e.g., positive prime–positive target), processing is facilitated and results in shorter reaction times, whereas if it is of different valence (e.g., positive prime–negative target), processing is inhibited and followed by longer reaction times (Hermans et al., 1994).

Accordingly, to further elucidate the relationship between arithmetic, emotions and low achievement, Rubinsten and Tannock (2010) investigated the effects of mathematics anxiety on numerical processing using a novel affective priming task. Their task differed from that typically utilized in standard affective priming procedures, in that not only positive, negative and neutral primes but also mathematics-related ones were included. The assumption was that this arithmetic-affective priming task acts as an indirect measure of mathematics anxiety. The sample consisted of 23 participants (12 children with DD and 11 control children) that were all above grade 4. Children had to complete a priming task where a priming word was followed by an arithmetic operation. They then had to indicate whether the arithmetic operation was true or false. As mentioned above, in priming tasks the priming word often cannot be ignored by participants, which is why it interferes with arithmetic performance. The priming words presented were either with a positive, negative or neutral affect or with some relation to mathematics. The arithmetic operations included were single-digit additions, subtractions, multiplications or divisions. As hypothesized, a direct link appeared between emotions (primes) and the arithmetic operations, and this association was different in children with DD and controls. Precisely, children with DD responded faster when the preceding priming word was negative or mathematics-related. This is in line with the assumption of affective priming paradigms that if the prime-target pair is of the same valence, processing is facilitated and hence faster. This implies that for DD children, mathematics-related primes are as negatively attributed as negative primes themselves. In the control children a reversed pattern was observed since mathematic related primes inhibited processing. Based on their findings, Rubinsten and Tannock concluded that a negative math priming effect exists in children with DD and that the arithmetic-affective priming task could be used as an indirect measure of mathematics anxiety in these children.

The current study aimed to reinvestigate the finding that in children with DD mathematics-related and negative primes have a similar effect on performance, particularly a facilitative influence on arithmetical processing. This analogous influence of negative affective and mathematics-related primes is henceforth referred to as the *negative math priming effect*. To enable an in-depth study of the relation between mathematics, emotions and performance, a large number of children ranging in age from 7.3 to 11.3 years was examined by detailed neuropsychological assessments and an adapted version of the priming task by Rubinsten and Tannock (2010). Notably, in addition to the indirect measure of mathematics anxiety, we also included a direct measure, namely the Math-Anxiety-Interview (please see section Cognitive assessments). Accordingly, the present study addresses the above-mentioned general lack of research in younger children and provides new insights into direct and indirect measures of the relation between mathematical performance and emotions.

## Materials and methods

### Subjects

A total of 183 children were recruited and their parents agreed to participate. The aim was that approximately half of the children had a diagnosis of DD and the other half with typical development to achieve equally sized groups. Of the 183 children, 172 children (aged 7.3–11.3 years, mean 8.6 years, 69.8% female) met the general inclusion criteria and hence comprised the final study sample. 76 children (44.2%) further met the criteria for DD, the other 96 children served as control children (CC) (see also Table 1 for demographic and behavioral data). The children were recruited in Germany (Berlin, Potsdam) and Switzerland (Zurich).

**Table 1:** Demographic and behavioral data of the sample.

	**Total**	**DD**	**CC**	**Statistics**
Subjects (N)	172	76	96	
Age (years) *M* (*SD*)	8.59 (0.95)	9.04 (1.03)	8.23 (0.71)	*U* = 1863.5, z = −5.51, *p* < 0.001
Gender (male/female)	52/120	22/54	30/66	χ^2^(1, N = 172) = 0.11, n.s.
Intelligence (IQ)^a^ *M* (*SD*)	101.06 (7.69)	96.53 (5.73)	104.65 (7.16)	*U* = 1402.5, *z* = −6.92, *p* < 0.001
Mathematical performance (*T*)^b^ *M* (*SD*)	43.83 (9.39)	34.99 (3.65)	50.83 (6.00)	*U* < 0.001, *z* = −11.25, *p* < 0.001
Math anxiety (intensity)^c^ *M* (*SD*)	3.24 (2.52)	4.37 (2.41)	2.35 (2.24)	*U* = 1909, *z* = −5.37, *p* < 0.001
Arithmetic fluency (T)^d^ *M* (*SD*)	41.60 (10.13)	33.35 (5.71)	48.13 (7.84)	*t*(169) = 14.29, *p* < 0.001
Addition (% correct)^e^ *M* (*SD*)	81.66 (20.72)	68.31 (23.65)	92.30 (8.53)	*U* = 1058, *z* = −7.30, *p* < 0.001
Subtraction (% correct)^e^ *M* (*SD*)	71.53 (23.57)	54.51 (22.76)	85.11 (13.08)	*U* = 812.5, *z* = −8.09, *p* < 0.001
Number line (% deviation)^e^ *M* (*SD*)	4.73 (2.64)	5.77 (3.03)	3.91 (1.92)	*U* = 1977, *z* = −4.06, *p* < 0.001
Number line (*T*)^f^ *M* (*SD*)	55.95 (14.65)	47.19 (12.71)	62.87 (12.2)	*U* = 1226, *z* = −7.47, *p* < 0.001
Working memory (items)^g^ *M* (*SD*)	4.05 (1.65)	3.77 (1.58)	4.28 (1.68)	*U* = 3184.5, *z* = −1.12, n.s.

a *Mean IQ based on 4 subtests [verbal IQ and matrices test of BUEGA, block design and similarities subtest of WISC-IV (n = 153)], or based on 6 subtests of the WISC-IV [block design, similarities, digit span, picture concepts, vocabulary, arithmetic (n = 19)]*.

b *Mean mathematical performance based on 4 subtests (addition and subtraction of HRT, Zahlenstrahl II of ZAREKI-R, Rechentest of BUEGA) (n = 153)], or based on addition and subtraction of HRT and ZAREKI-R (n = 19) in T-values*.

c *Mean intensity of math anxiety assessed by the math anxiety interview (MAI); 0 = no math anxiety, 10 = very high math anxiety*.

d *Mean of addition and subtraction of the HRT in T-values*.

e *Based on number line task. The percentage of correctly solved addition or subtraction problems are listed. Moreover, the percentage of the deviation between the exact location on the number line and the marked location of the child is indicated*.

f *Based on subtests number line I and II of ZAREKI-R in T-values*.

g *Based on maximum number of correctly recalled items of the Corsi-Suppression test*.

#### General inclusion/exclusion criteria

Of the originally 183 children, a total of 11 children (6%) were excluded from the data analyses due to the following criteria: Three children were excluded because their Intelligence Quotient (IQ) was above 120. Seven children were excluded due to psychiatric diagnoses. One child was excluded due to undefined group membership (with mathematical performance *T* = 40.44, see criteria for classification of DD).

Parents gave written consent and children received a voucher for their participation. The study was approved by the local ethics committee based on guidelines from the World Medical Association's Declaration of Helsinki (WMA, 2002).

#### Classification of DD

Classification of DD was based on the diagnostic criteria of the DSM-V (APA, 2013): criterion (A) severe math problems for more than 6 months; criterion (B) Low achievement scores in one or more standardized mathematical tests (1–2.5 SD below the population mean for age); criterion (C) mathematical difficulties readily apparent in the early school years; criterion (D) not attributable to intellectual disabilities (IQ > 70), global developmental delay, hearing or vision disorders, or neurological or motor disorders. In the present study, mathematical performance was assessed in all children by a careful selection of standardized neuropsychological tests particularly designed for the clinical assessment of DD (for detailed description see section Cognitive Assessments). To be classified as having DD, the mean *T*-value of mathematical performance had to be lower than 40 (<1 SD). In addition, general intelligence had to be in the normal range (85 < IQ < 114, *N* = 173; or marginally above IQ 115–120, *N* = 7) with no evidence of any psychiatric disease. According to DSM-V, the discrepancy between mathematical performance and individual IQ is not a requirement for diagnosis, nevertheless, 78% of the DD children showed a discrepancy between mathematical performance and IQ of more than one standard deviation (*N* = 59, mean discrepancy = 14.3 *t*-value), and 22% showed a lower discrepancy between both measures (*N* = 17, mean discrepancy = 7.1 *t*-value).

### Cognitive assessments

#### Intelligence

Estimated intelligence was measured by the mean of different IQ subtests. Mean IQ of children recruited in Switzerland was based on six subtests of the standardized Wechsler Intelligence Scale for Children (WISC-IV) test battery (block design, similarities, digit span, picture concepts, vocabulary, and arithmetic) (Petermann and Petermann, 2007). The mean IQ of children recruited in Germany was based on four subtests, including two of the WISC-IV (block design, similarities) and two of the test battery “Basisdiagnostik Umschriebener Entwicklungsstörungen im Grundschulalter” (BUEGA) (Esser et al., 2008).

#### Mathematical performance

The main mathematical and numerical performance for children recruited in Switzerland was assessed using the two subtests of the Heidelberger Rechentest (HRT) (addition, subtraction) (Haffner et al., 2005) and the standardized Neuropsychological Test Battery for Number Processing and Calculation in Children (ZAREKI-R) (von Aster et al., 2006). This neuropsychological battery examines basic skills in calculation and arithmetic and aims to identify and characterize the profile of mathematical abilities in children with DD from the 1st to 4th grade level. It is composed of 11 subtests, such as reverse counting, subtraction, number reading, dictating, visual estimation of quantities, and digit span forward and backward. Mean number processing for children recruited in Germany was assessed using four subtests, namely two of the Heidelberger Rechentest (HRT) (addition, subtraction) (Haffner et al., 2005), the number line task II of the ZAREKI-R and the calculation test of the BUEGA (Esser et al., 2008). The calculation test of the BUEGA evaluates by text problems, which are illustrated in pictures, the knowledge of number comparisons, magnitudes, sizes, and the understanding and use of the four basic arithmetical operations. Criteria for DD for both Swiss and German children were met if a child's performance was below a mean *T*-value of 40.

#### Mathematics anxiety

Mathematics anxiety was assessed by the Math-Anxiety-Interview for German speaking primary school children (MAI), which is a valid and reliable measure for the assessment of math anxiety as demonstrated by a Cronbach's alpha of 0.90 (Kohn et al., 2013). The MAI combines two different types of questions while four math related situations are verbally and pictorially presented (1st on the eve of a math test, 2nd math homework, 3rd math class, and 4th everyday/shopping). The child is initially asked to rate the intensity of his or her anxiety concerning the presented situation by an anxiety thermometer from 0 to 10. In a second step, the different components of anxiety (affective, cognitive, behavioral and physiological) are explored. The child is asked to estimate, to what extent specific statements apply to the particular situation, e.g., “I cannot get a word out.” For the present study we have chosen the mean math anxiety intensity associated with all four situations which provides a valid and reliable measure from 0 = no anxiety to 10 = very strong math anxiety in primary school children.

#### Arithmetic fluency

Arithmetic fluency was evaluated using the addition and subtraction subtests of the Heidelberger Rechentest (HRT). In this test, a list of 40 addition or subtraction tasks is presented to the child and he/she is asked to solve as many problems as possible within 2 min.

#### Number line performance

The spatial representation of numbers was measured by a paper-and-pencil number line task (Kucian et al., 2011). Children had to indicate with a pencil on a left-to-right oriented number line from 0 to 100 the location of 20 Arabic digits, the results of 20 additions and 20 subtractions, and the estimated number of 10 different dot arrays. The accuracy was measured by calculating the percentage distance from the marked to the correct position of the given number (=%deviation). Only the correctly calculated addition and subtraction problems were included, but the percentage of correctly solved addition or subtraction trials was also calculated.

#### Working memory

Spatial working memory was assessed by the Block Suppression Test (Beblo et al., 2004). This test is based on the CORSI-Block Tapping test (Schellig, 1997) and requires the subject to reproduce every second block in a given sequence of touched cubes on a wooden board as the examiner demonstrated. While the sequences gradually increase in length, the number of cubes last tapped in two consecutively correct sequences is defined as the maximum spatial working memory span.

### Priming task

**Figure 1:**
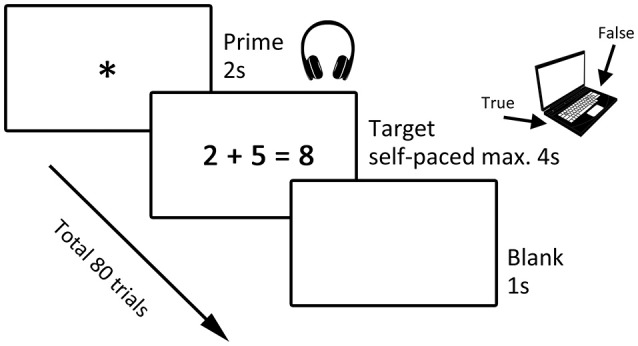
Paradigm. The paradigm consisted of a priming task including a prime and a target. Primes were either positive, negative, neutral or mathematics-related and were presented aurally while children focused on a fixation star for 2 s. Then a subtraction or addition problem followed as the target and children had to inadequate whether it was true or false by pressing either button p or q on the keyboard. This presentation of the target was self-paced with a maximum of 4 s. The trial ended with the presentation of a blank screen for 1 s.

To assess the affective effects of primes on calculation an adapted version of the task developed by Rubinsten and Tannock (2010) was used. It included four different types of primes (words with either positive, negative or neutral affect and words related to mathematics) and single-digit arithmetic problems (additions and subtractions) served as targets. As illustrated in Figure 1, each trial consisted of a prime presented aurally via headphones followed by an arithmetical problem. Reaction time in milliseconds was measured by the computer from the target onset to the participant's response. Each participant underwent in total 80 trials.

The primes were comprised of 40 words, including 10 per affective dimension (e.g., sun as a positive affective word, wood as a neutral affective word, prison as a negative affective word and count as a mathematic related word). The words were selected from the “The Berlin Affective Word List Reloaded” (BAWL-R), which contains a large set of psycholinguistic indexes known to influence word processing, and also features ratings of the emotional arousal, emotional valence and imageability of each word (Võ et al., 2009). Since the word ratings of the BAWL-R were based on ratings from 200 adults, we selected 61 words, which were balanced for the number of letters, number of syllables, type of word (noun, verb, adjective), and emotional valence (positive, negative, neutral) or with mathematics related content and had them re-evaluated by a group of children. In total 123 children from the 2nd (*N* = 29), 3rd (*N* = 30), 4th (*N* = 32), and 5th (*N* = 32) grade rated these words. They were asked to indicate how they felt when they heard the word by marking a five-stepped smiley scale from happy to sad smileys, and to indicate if the word was related to mathematics, yes or no. Obtained ratings were analyzed for all children, as well as for each grade and for girls or boys separately. Consideration of these findings led to the final word list that was used in the present priming task. Please see Table S7 for a detailed description of the words.

The arithmetic problems were presented in the form “a ^*^ b = c,” where a and b represent single digits from 1 to 9, ^*^ represents an arithmetic operation (+ or –) and c represents the solution, which also consisted of only one digit (e.g., 2 + 1 = 3 as a correct addition target). The type of arithmetic operation (each prime was once followed by an addition and once by a subtraction problem) and whether the problem was true or false were balanced between affective dimensions of primes and presented in a randomized order.

The present task differed from the original one of Rubinsten and Tannock (2010) in the following aspects: We have simplified the task by reducing the total number of trials from 160 to 80 by excluding multiplication and division arithmetic problems. Furthermore, the maximal response latency was extended from 3,000 to 4,000 ms. Moreover, we only included single digit solutions. Whereas Rubinsten and Tannock presented the primes visually in English, we presented the primes aurally in German. These changes in the priming task were conducted to adapt the paradigm to our younger cohort, consisting mostly of children in grades 2 or 3, whereas subjects in the study of Rubinsten and Tannock were in grades 4 or above.

### Data analyses

Data were analyzed by IBM SPSS Statistics Version 24 (IBM SPSS Statistics for Windows, 2016). Raw data of the priming task were extracted from E-Prime (E-Prime, 2002) and converted into SPSS. First, all behavioral data were tested for normality by the Kolmogorov-Smirnov test. If the data followed a normal distribution, groups were compared by parametric independent-sample *t*-tests. If data were not normally distributed, the nonparametric Mann-Whitney *U*-test for two independent samples was used. Nominal data (gender) was compared between control children and the DD group with a chi-squared test. *P*-values lower than 0.05 were considered statistically significant. To evaluate the effects of the priming task a general linear model analysis was conducted with RT as dependent variable. The 4 (type of prime: positive/negative/neutral/mathematics-related) × 2 (arithmetic operation: addition/subtraction) repeated measures ANCOVA defined type of prime and arithmetic operation as within-subject factors and group (CC/DD) as the between-subject factor. Since DD and CC groups differed in age, age was included as a covariate to exclude the possibility that group differences might be based on age differences. Regarding IQ, it was expected that children with DD show a lower mean IQ compared to typically achieving peers as IQ measures are not fully independent from measures of math ability (Lambert and Spinath, 2018). In our analyses, we decided not to match groups on IQ, because one would have artificially influence the pattern of the normal population of DD or CC children. Moreover, IQ not to include as covariate in statistical analyses, which is in line with the suggestion of Dennis et al. (2009), who state that it is misguided and unjustified to attempt to control for IQ differences for cognitive outcome. However, we repeated the all tests with IQ and age as covariates showing that the main results did not change.

## Results

### Demographic and behavioral data

Findings of demographic and behavioral data are summarized in Table 1. Statistical group comparisons indicated that children with DD were significantly older compared to control children, but groups did not differ in gender distribution. As expected, children with DD performed worse in all mathematical tests (addition and subtraction subtests of HRT, subtests of ZAREKI-R, Rechentest of BUEGA, and number line task). Even though all children had an IQ in the normal range, the IQ of the control group was significantly higher. In visuospatial working memory, no group differences were evident.

General findings regarding accuracy and reaction time between groups (CC/DD) or between conditions (addition/subtraction) are displayed in Table 2. For accuracy, the group comparison revealed significant differences between control and DD children in total accuracy and the accuracy of addition or subtraction problems separately, such that control children made significantly fewer errors. Furthermore, all children showed a higher accuracy for addition compared to subtraction (all children *N* = 172, *Z* = −5.29, *p* < 0.001; DD, *N* = 76, *Z* = −3.56, *p* < 0.001; CC, *N* = 96, *Z* = −3.96, *p* < 0.001). Results indicated that all participants were faster for addition problems compared to subtraction problems [all children *N* = 172, *t*_(170)_ = −4.96, *p* < 0.001; DD, *N* = 76, *t*_(75)_ = −2.26, *p* < 0.05; CC, *N* = 96, *t*_(94)_ = −4.62, *p* < 0.001], but no significant differences between control children or dyscalculic children were found (please see Table 2).

**Table 2:** General findings on accuracy and reaction time.

	**Total**	**DD**	**CC**	**Statistics between groups**
Subjects (N)	172	76	96	
Accuracy (%)^a^ *M* (*SD*)	72.71 (18.10)	66.33 (18.19)	77.76 (16.43)	*U* = 2236, *z* = −4.36, *p* < 0.001
Accuracy addition (%)^a^ *M* (*SD*)	75.23 (19.06)	69.47 (19.62)	79.79 (17.40)	*U* = 2446.5, *z* = −3.71, *p* < 0.001
Accuracy subtraction (%)^a^ *M* (*SD*)	70.23 (19.00)	63.29 (18.95)	75.72 (17.24)	*U* = 2180, *z* = −4.53, *p* < 0.001
Reaction time^b^ (ms) *M* (*SD*)	2278.34 (492.26), *N* = 169	2239.94 (544.05), *N* = 75	2308.97 (447.30), *N* = 94	*t* (142) = 0.89, n.s.
Reaction time additions^b^ (ms) *M* (*SD*)	2200.77 (412.23), *N* = 172	2225.68 (420.53), *N* = 76	2181.05 (406.67), *N* = 96	*t* (170) = −0.70, n.s.
Reaction time subtractions^b^ (ms) *M* (*SD*)	2287.24 (393.14), *N* = 171	2282.29 (384.45), *N* = 76	2291.19 (401.95), *N* = 95	*t* (169) = 0.15, n.s.

a *Mean accuracy of all 80 trials in %, or respectively of 40 trials for addition or subtraction*.

b *Mean reaction time including only correct trials within the range of ± 2.5 SD from the conditional mean latency in milliseconds (ms), for all, or only addition or subtraction problems*.

### Affective priming

**Figure 2:**
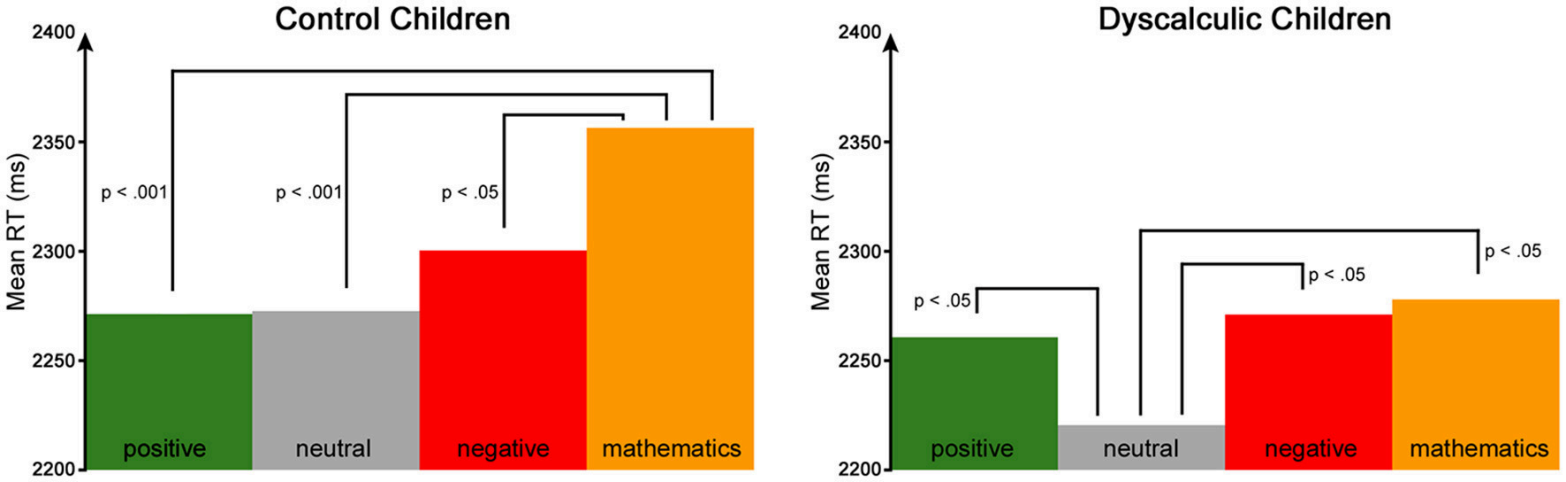
Affective priming results. The figure presents mean RT for positive, neutral, negative and mathematics-related primes for control children **(left)** and DD **(right)**.

To analyze potential *negative math priming effects*, a repeated measures ANCOVA was used. The type of prime (positive/negative/neutral/mathematics-related) and arithmetic operation (addition/subtraction) were defined as within-subject factors, group (CC/DD) was defined as the between-subject factor and age as a confounding variable. The analysis revealed no significant main effects for the type of prime [*F*_(3, 157)_ = 1.087, n.s.] or arithmetic operation [*F*_(1, 159)_ = 0.149, n.s.]. However, the interaction between type of prime and group reached significance [*F*_(3, 157)_ = 3.762, *p* = 0.012, η^2^ = 0.067]. This interaction was further analyzed with *post-hoc* tests separated by group. As displayed in Figure 2 and Table 3, in the *control children*, response latencies were significantly shorter for positive, negative and neutral primes compared to mathematics-related primes. In the *dyscalculic children*, response latencies were significantly shorter for neutral than for positive, negative or mathematics-related primes. Hence, neither control children nor children with DD revealed a *negative math priming effect* as reported by Rubinsten and Tannock (2010).

**Table 3:** Reaction times (RT) as a function of the prime valence in control children (CC) and in children with developmental dyscalculia (DD).

**Prime valence**	**CC**						**DD**					
	**RT difference (ms)**			***df***	***t***	***p***	**RT difference (ms)**			***df***	***t***	***p***
	**N**	***Mean***	***SD***				**N**	***Mean***	***SD***			
Pos—Neg	93	−29.06	248.82	92	−1.13	n.s.	74	9.70	331.84	73	0.25	n.s.
Pos—Neutr	93	12.73	223.59	92	0.55	n.s.	73	83.41	270.05	72	2.64	<0.05
Pos—Math	93	−88.65	228.52	92	−3.74	<0.001	74	4.82	291.79	73	0.14	n.s.
Neg—Neutr	93	41.79	263.03	92	1.53	n.s.	72	71.51	298.82	71	2.03	<0.05
Neg—Math	93	−59.58	257.92	92	−2.23	<0.05	73	−2.61	290.75	72	−0.08	n.s.
Neutr—Math	94	−94.35	250.39	93	−3.65	<0.001	72	−78.72	287.65	71	−2.32	<0.05

Although we found no main effect of arithmetic operation in the present study, we also analyzed the effects of primes separately for addition and subtraction problems to allow direct comparison to the results of Rubinsten and Tannock (2010); please see Supplementary Material 1. Affective priming split by arithmetic operations, including Table S1). Similar to our main findings, no effects of prime were found. Regarding the arithmetic operation, reaction times (= *dependent variable*) were further contrasted for the groups depending on the type of prime. While in the *control group* significantly shorter response latencies were found for addition than for subtraction across all type of primes, in the *DD group* no significant differences were evident. Please see Supplementary Material 2. Differences between arithmetic operations split by primes, including Table S2).

The entire analysis was also performed after only including children who performed above chance level (mean accuracy ≥50%). Results of the repeated measures ANCOVA indicated no significant main effects or interactions. However, it is noteworthy that the interaction between type of prime and group missed significance only narrowly [*F*_(3, 142)_ = 2.654, *p* = 0.051, η^2^ = 0.053]. For a detailed description of the demographic and behavioral data for this subgroup of children, please see Supplementary Material 3. Data analyses for accuracy levels above chance, including Table S3.

### Gender differences

In general, boys and girls tend to have different attitudes to mathematics such that girls express more concern about their mathematical performance (reviewed by Johns et al., 2005). Previous reports that girls show more mathematics anxiety (reviewed by Dowker et al., 2016), and that this math anxiety had an effect on mathematical performance lead us to the analyses of possible gender differences. Moreover and especially with regard to the affective priming task, it is important to note that gender differences have been reported in emotion processing too (reviewed by Hamann, 2004). Hence, for a more detailed understanding of possible gender differences in priming effects, analyses were performed after splitting the control and DD children into male and female subgroups. All results are presented first for the control and then for the DD children. For demographic and behavioral data please see Supplementary Material 4. Gender differences, including Tables S4, S5. In sum, control boys performed significantly better in arithmetic fluency, whereas this pattern was reversed in the DD children, and DD girls additionally performed better in working memory.

Boys and girls were then analyzed for priming effects. A repeated measures ANOVA was used in which type of prime (positive/negative/neutral/mathematics-related) and arithmetic operation (addition/subtraction) were defined as within-subject factors and gender as between-subject factor.

In *control children*, significant main effects for type of prime [*F*_(3, 88)_ = 7.977, *p* < 0.001, η^2^ = 0.214] and arithmetic operation [*F*_(1, 90)_ = 27.888, *p* < 0.001, η^2^ = 0.237] were found, but no gender effects were observed. For *post-hoc t*-tests regarding prime or operation please see Tables 2, 3. In *dyscalculic children*, the repeated measures ANOVA revealed significant main effects for type of prime [*F*_(3, 66)_ = 6.225, *p* = 0.001, η^2^ = 0.221] and operation [*F*_(1, 68)_ = 5.192, *p* = 0.026, η^2^ = 0.071], but again no gender effects. Results of *post-hoc* tests for the effect of prime and operation are summarized in Tables 2, 3.

Taken together, no gender effects were evident in DD or control children and hence no gender-dependent *negative math priming effects* were found.

### Mathematics anxiety

The analyses were also repeated after taking into account the children's level of mathematics anxiety, quantified through the direct, explicit measure of the Math-Anxiety-Interview (please see section Cognitive Assessments). This complements the implicit measure of mathematics anxiety through the priming task. The math anxiety interview was performed because it is known that mathematics anxiety has an impact on mathematical performance (reviewed by Wu et al., 2014). Hence, the performance in the task may not solely be influenced by the affective priming, but also by the extent of children's mathematics anxiety. Two different analyses with regard to mathematics anxiety were performed: firstly, a bivariate correlation was calculated between the level of mathematics anxiety (MAI) and the difference in reaction times between mathematics-related and negative affective primes. This was in order to test whether there exists a relationship between math anxiety and a possible *negative math priming effect*. As both variables were not normally distributed, a Spearman Correlation was carried out, which, however, was not significant (*r*_*s*_ = −0.057, *N* = 172, n.s.).

Secondly, to further analyse potential effects of mathematics anxiety, the sample was split according to different levels of mathematics anxiety (low vs. high). Hence, two new groups were formed by selecting the 25% of children with the lowest or highest MAI values, respectively. The lowest 25% of children had a MAI-value of 0-1 and the highest 25% of children had a MAI-value of 5.9-9.75. In order to avoid having to select between children with the same MAI-value, all children with the respective threshold value were included. That is the reason why samples are not perfectly equal in size, resulting in a total number of *N* = 46 in the low math anxious group and N = 54 in the high math anxious group. Demographic and behavioral data is included in the Supplementary Material 5. Mathematics anxiety, Table S6. In summary, both groups were matched for age and gender, but the high anxious subgroup included more DD children (72% vs. 26%). Accordingly, the high anxious group performed worse in different mathematical and general cognitive tasks.

The two groups were then further analyzed for priming effects of reaction time as the dependent variable. A repeated measures ANCOVA was used with type of prime (positive/negative/neutral/mathematics-related) and arithmetic operation (addition/subtraction) as within-subject factors and low vs. high mathematics anxiety as between-subject factor. Group (CC/DD) was included as covariate to control for unequal distribution of DD and control children in low vs. high mathematics anxiety subgroups. The main effect of prime [*F*_(3, 88)_ = 3.695, *p* = 0.015, η^2^ = 0.112] and the interaction between prime and group (low vs. high anxious) reached significance [*F*_(3, 88)_ = 3.389, *p* = 0.022, η^2^ = 0.104]. Hence, the interaction was further investigated separately for the low and high mathematics anxiety group.

As illustrated in Table 4, *post-hoc* tests showed that in the *low anxious children*, response latencies were significantly shorter for positive than for mathematics-related affective primes. Furthermore, response latencies were significantly shorter for neutral than for negative or math affective primes. In *high anxious children*, no differences in response latencies to the different primes were observed. In sum, no *negative math priming effects* were evident for low or high anxious children. These findings are comparable with the results when groups were split by DD and CC, where the results of the low anxious children reflect findings of CC group.

**Table 4:** *Post-hoc* tests for type of prime (prime valence) in low vs. high mathematics anxiety (MAI) children.

**Prime valence**	**Low MAI group**						**High MAI group**					
	**RT difference (ms)**			***df***	***t***	***p***	**RT difference (ms)**			***df***	***t***	***p***
	**N**	***Mean***	***SD***				**N**	***Mean***	***SD***			
Pos—Neg	44	−66.38	223.49	43	−1.97	n.s.	51	−5.09	342.77	50	−0.11	n.s.
Pos—Neutr	44	10.94	206.48	43	0.35	n.s.	50	40.32	291.76	49	0.98	n.s.
Pos—Math	44	−104.90	251.27	43	−2.77	<0.05	52	−20.08	330.45	51	−0.44	n.s.
Neg—Neutr	44	77.32	222.41	43	2.31	<0.05	49	41.90	296.25	48	0.99	n.s.
Neg—Math	44	−38.52	303.73	43	−0.84	n.s.	51	−24.84	302.90	50	−0.59	n.s.
Neutr—Math	44	−115.84	233.90	43	−3.29	<0.05	51	−60.80	320.37	50	−1.36	n.s.

In addition to possible effects of math anxiety on priming, we further investigated general characteristics of math anxiety which were explicitly evaluated by the MAI. First, as listed in Table 1, children with DD suffer more often from math anxiety. Second, no gender differences were evident in CC (see Table S4) or in DD (see Table S5). Third, Pearson correlation between math anxiety and age revealed no relation between both measures when including all subjects (*r* = 0.055, *N* = 172, n.s.), however, in DD children a decrease of math anxiety over development was found (*r* = −0.274, *N* = 76, *p* < 0.05), but not in CC (*r* = 0.030, *N* = 96, n.s.). Finally, the relation between math anxiety and behavioral measures was further investigated by Pearson correlations (please see Table 5). Including all children, results indicated a significant relation between math anxiety and IQ, mathematical performance, arithmetic fluency, addition, subtraction, number line performance, as well as working memory. In DD children, math anxiety was significantly related to mathematical performance, arithmetic fluency, addition, and subtraction. In CC, math anxiety correlated significantly with mathematical performance, arithmetic fluency, addition, subtraction, and number line performance. All these relations demonstrate that higher levels of math anxiety were associated with worse performance.

**Table 5:** Pearson's correlation between math anxiety and behavioral measures.

	**Math anxiety (intensity)^a^**		
	**Total**	**DD**	**CC**
Intelligence (IQ)^b^	*r* = −0.329 *p* < 0.001 *N* = 172	*r* = −0.158 n.s. *N* = 76	*r* = −0.150 n.s. *N* = 96
Mathematical performance (*T*)^c^	*r* = −0.508 *p* < 0.001 *N* = 172	*r* = −0.340 *p* < 0.01 *N* = 76	*r* = −0.368 *p* < 0.001 *N* = 96
Arithmetic fluency (*T*)^d^	*r* = −0.523 *p* < 0.001 *N* = 172	*r* = −0.344 *p* < 0.01 *N* = 76	*r* = −0.395 *p* < 0.001 *N* = 96
Addition (% correct)^e^	*r* = −0.531 *p* < 0.001 *N* = 160	*r* = −0.531 *p* < 0.001 *N* = 71	*r* = −0.267 *p* < 0.05 *N* = 89
Subtraction (% correct)^e^	*r* = −0.542 *p* < 0.001 *N* = 160	*r* = −0.456 *p* < 0.001 *N* = 71	*r* = −0.393 *p* < 0.001 *N* = 89
Number line (% deviation)^e^	*r* = 0.313 *p* < 0.001 *N* = 160	*r* = 0.196 n.s. *N* = 71	*r* = 0.230 *p* < 0.05 *N* = 89
Number line (*T*)^f^	*r* = −0.284 *p* < 0.001 *N* = 172	*r* = −0.071 n.s. *N* = 76	*r* = −0.110 n.s. *N* = 96
Working memory (items)^g^	*r* = −0.218 *p* < 0.01 *N* = 169	*r* = −0.187 n.s. *N* = 75	*r* = −0.163 n.s. *N* = 94

a *Mean intensity of math anxiety assessed by the math anxiety interview (MAI); 0 = no math anxiety, 10 = very high math anxiety*.

b *Mean IQ based on 4 subtests [verbal IQ and matrices test of BUEGA, block design and similarities subtest of WISC-IV (n = 153)], or based on 6 subtests of the WISC-IV [block design, similarities, digit span, picture concepts, vocabulary, arithmetic (n = 19)]*.

c *Mean mathematical performance based on 4 subtests (addition and subtraction of HRT, Zahlenstrahl II of ZAREKI-R, Rechentest of BUEGA) (n = 153), or based on addition and subtraction of HRT and ZAREKI-R (n = 19) in T-values*.

d *Mean of addition and subtraction of the HRT in T-values*.

e *Based on number line task. The percentage of correctly solved addition or subtraction problems, and the percentage of the deviation between the exact location on the number line and the marked location of the child*.

f *Based on subtests number line I and II of ZAREKI-R in T-values*.

g *Based on maximum number of correctly recalled items of the Corsi-Suppression test*.

## Discussion

Mathematics is often associated with negative attitudes and emotions in children and adolescents. However, little is known about the interactions between mathematical performance and negative emotions. Hence, this research gap was addressed by the present study. The aim was to elucidate the link between mathematics anxiety, negative emotions, low performance and deficiencies in mathematics abilities such as in children with DD.

To approach this question, an arithmetic-affective priming task was used in which the influence of a prime stimulus (positive/negative/neutral or mathematics-related) on a simple arithmetic operation (addition/subtraction) was tested in 172 children between 7.3 and 11.3 years of age. Approximately half of the children were diagnosed with DD.

Findings revealed, in line with our expectations, that all children were faster and made less errors for addition problems compared to subtraction. This is because subtractions often need more decomposing into smaller sub-parts and moreover, compared to additions, they are not commutative (e.g., 2 + 3 ≠ 3 – 2) (reviewed by Peters and De Smedt, 2017).

DD children performed worse in all mathematical tests and showed higher levels of mathematics anxiety, which remained significant when controlling for age, since the CC were slightly younger than the DD children. This is important to note, as it has been often been claimed, but so far only few studies examined and corroborated the increased levels of mathematics anxiety in subjects with disabilities in mathematics (Wu et al., 2014). Moreover, children with DD often suffer from additional psychiatric disorders like general anxieties (reviewed by Dowker et al., 2016).

Regarding gender differences, our results indicated that girls and boys of the CC or DD group experienced math anxiety equally often. This is a promising result regarding the widely discussed stereotype that females are expected to be worse in math related topics and that females experience more math anxiety. Our findings are consistent with research indicating that countries providing equal education for females and males show little or no gender differences in mathematical performance (Spelke, 2005; Kohn et al., 2013). The reason why no gender differences in math anxiety were evident might be due to increasing evidence that such gender differences only develop at adolescence as consequence of societal exposure to gender stereotypes (e.g., Johns et al., 2005), or female teachers who experience math anxiety themselves (Beilock et al., 2010). In contrast, several studies report that younger children in primary school do not exhibit gender differences in math anxiety (e.g., Dowker et al., 2012; Harari et al., 2013). Our findings are consistent with these reports since the children in our study were in primary school.

In general, studies suggest that math anxiety increases with age (reviewed by Dowker et al., 2016). The present study rather suggests that math anxiety is already present in 8-year old children, which is consistent with reports suggesting that math anxiety can be detected in the earliest stages of formal math learning in school (Wu et al., 2012; Sorvo et al., 2017). In addition, math anxiety in children with DD even seemed to decrease over development, which might be a positive effect of increased care, but would need specific investigation.

A large body of evidence confirms that math anxiety severely interferes with math learning and performance, both because math anxious people are more likely to avoid mathematical activities and because math anxiety usurps working memory resources (reviewed by Dowker et al., 2016). Similarly, the present findings also revealed that children with increased math anxiety performed worse in math related topics (mathematical performance, arithmetic fluency, addition, subtraction, number line performance), as well as, working memory. Since the majority of our children with math anxiety belonged to the DD group, the present findings are in line with the notion that DD children show specific deficits in visuospatial working memory, but it is not possible to further differentiate between effects of math anxiety or DD on different working memory profiles (Mammarella et al., 2015). Moreover, a significant relationship between math anxiety and IQ was found. In terms of domain-general abilities, it has been suggested that poor intellectual conditions (e.g., poor abstract thinking or poor visuospatial skills) may contribute to the development of math anxiety (reviewed by Suárez-Pellicioni et al., 2016). However, the relation between math anxiety and IQ might also be a result of the group composition in the present study and can be rather attributed to the relation between math anxiety and math performance, since within groups (DD or CC) no relation between math anxiety and IQ was found.

With regard to priming effects however, the present data did not corroborate results reported by Rubinsten and Tannock (2010). Since no differences were found in reaction times to positive or negative affective primes, no standard affective priming effects were evident in control or DD children. Furthermore, no *negative math priming effect* was found in DD children. Thus, mathematics-related primes did not act affectively related to targets. In the study by Sarkar et al. (2014), the priming paradigm from Rubinsten and Tannock (2010) was adopted, however, no mathematics-related words were included and hence no direct comparison is possible between their results and our main finding regarding the *negative math priming effect*. Nevertheless, no significant effects of valence (positive or negative primes) were found, which is in line with our finding of absent standard priming effects.

In our analysis, no main effects of group, type of prime or arithmetic operation were evident. However, we found a significant interaction between type of prime and group, indicating that the reaction to positive, negative, neutral and mathematics-related primes is significantly different between DD and control children.

Overall, our major result is that we did not find the *negative math priming effect* observed by Rubinsten and Tannock (2010). In contrast, our analysis showed that control children's responses were significantly faster after the presentation of a positive, neutral or negative affective prime compared to a mathematics-related one. DD children were significantly faster after a neutral affective prime compared to a positive, negative or a mathematics-related one. This implies that control children's performance was inhibited by mathematics-related primes but was not affected differently by positive, neutral or negative primes. In contrast, DD children's performance was facilitated if the presented prime was neutral. Taken together, mathematics-related primes significantly interfered with processing the target in control children, but not in the DD group.

In conclusion, control children showed better performance if the prime had either an affective valence (positive or negative) or no valence (neutral), but not if there was a relation to mathematics. Thus, control children's processing seemed to be inhibited by mathematics-related primes. This might be a consequence of incongruent prime-target pairs. For example, if the mathematics-related prime word was “minus” and the subsequent target was an addition, this conflict might have disturbed processing in control children. It is implied that even if the prime word is instructed to be ignored, it automatically interferes with processing. This argument is supported by the Stroop effect, in which an interference is shown when processing an incongruent condition of font color and word meaning which results in slower reaction times (Stroop, 1935). Similar effects have been reported in the context of mathematics, hence called the numerical Stroop effect, in which the physical size of the presented number interferes with the judgment of actual number size (e.g., Kaufmann et al., 2005). Accordingly, it seems plausible that mathematics-related prime words could interfere with subsequent calculation processes but only in control children. Similarly, these congruity effects have been reported in healthy participants, whereas they were not evident or much smaller in subjects with DD (Rubinsten and Henik, 2005). As found in the present study, this lends further support to the notion that dyscalculic subjects, unlike typically developing children, fail to process the irrelevant dimension automatically. In contrast, DD children performed faster if the prime had no valence (neutral). This is in contrast to what is expected according to the results of affective priming paradigms. As mentioned, faster processing is hypothesized if the prime-target pair is of the same valence (Hermans et al., 1994), which can already be observed in children (Spence et al., 2006). Since we assume that mathematics is negatively associated in children with DD, processing was expected to be faster if the prime had a negative valence or was mathematics-related.

In sum, since reaction times to positive or negative affective primes did not differ, the present study could neither find standard affective priming effects in control nor in DD children. Importantly, reaction times to negative or mathematics-related primes did not differ either, which clearly shows that no *negative math priming effect* in DD children was evident. A closer look at Rubinsten and Tannock's study, which suggested a *negative math priming effect* in DD, revealed that they did not observe the *negative math priming effect* in subtraction and division either. While they did observe it in addition and multiplication, an opposite pattern was shown for subtraction and for division the authors reported even a more facilitating influence of mathematics-related primes compared to negative primes. The fact that we did not observe the *negative math priming effect* in the present study might therefore be due to pooling addition and subtraction during the task. Therefore, we further analyzed our data separately for addition and subtraction. In line with Rubinsten and Tannock (2010), we did not observe the *negative math priming effect* in subtraction trials. In contrast, the *negative math priming effect* was absent in addition in the present study too.

To further investigate the inconsistent findings in terms of the *negative math priming effect*, several additional analysis were conducted that considered accuracy levels, gender and mathematics anxiety. As the amount of errors differed substantially between Rubinsten and Tannock (2010) and the present study, we decided to re-analyze the data and apart from solely including correct responses, only children that performed above chance level were considered. Consistent with our main findings, even when considering accuracy levels, no *negative math priming effect* was observable. Hence, the absence of the *negative math priming effect* cannot be justified by overall lower accuracy levels of children in the present study.

Furthermore, we carried out an analysis including gender, as previous research assumed attitudes and levels of mathematics anxiety to be different in boys and girls (reviewed by Dowker et al., 2016). Hence, these differences were considered to affect priming effects. However, no priming effects were evident in boys or in girls, which again strengthens our major finding that we did not observe a *negative math priming effect*. Thus, gender is not a driving force of effects detected by Rubinsten and Tannock (2010). Regarding gender, it is also of interest that in our sample boys and girls of both the control and the DD group did not differ in mathematics anxiety. This is noteworthy as the literature reported inconsistent gender differences. Furthermore, math anxiety is supposed to increase with age during childhood and adolescence (reviewed by Dowker et al., 2016), which is a possible reason why no differences were evident in the present sample represented by rather young children. Similarly, empirical data including that from younger children also reported no differences in the levels of mathematics anxiety between boys and girls, since these differences only develop in adolescence (Johns et al., 2005).

Lastly, different levels of mathematics anxiety were taken into account as explanatory factors. The rationale underlying this analysis is the assumed negative influence of mathematics anxiety on mathematical performance (reviewed by Dowker et al., 2016). Hence, we hypothesized that priming effects could appear when groups were separated by extreme levels of mathematics anxiety. Concretely, this examination included a direct measure of mathematics anxiety (assessed by the MAI Kohn et al., 2013). This explicit measure of mathematics anxiety is an interesting add-on to our examination. Compared to Rubinsten and Tannock's implicit measure of mathematics anxiety through the priming paradigm, we consider the MAI to provide a more reliable assessment of mathematics anxiety. However, the potential relation between mathematics anxiety and the *negative math priming effect* was not significant. Furthermore, the analysis split into low vs. high anxious children found no *negative math priming effect* neither in the group of low, nor in the group of highly math anxious individuals. Therefore, mathematics anxiety is not causative for the priming effects observed by Rubinsten and Tannock (2010), which further supports our main result that no *negative math priming effect* was evident. Furthermore, these findings additionally support the notion that there is no relation between explicitly quantified math anxiety and the proposed implicit measure of math anxiety by the priming task. This leads to the conclusion that the priming task probably does not reliably assess math anxiety.

Taken together, further analyses showed that the *negative math priming effect* is independent of accuracy levels, gender, and mathematics anxiety, as it could still not be replicated when considering these variables. However, one might argue that present findings point toward an affective priming effect (comparing effects of positive and negative primes) since both the low math anxious subgroup and the control group reacted faster after a positive prime than after a negative prime. In the high math anxious subgroup and the DD group, this was not the case. This might hint to a possible positive valence of calculation problems in low math anxious or control children and a negative valence of arithmetical problems in high math anxious or DD children. Similarly, results seem indicative of a negative math priming effect (comparing effects of positive and mathematical primes) as both low math anxious individuals and control children are faster after positive than mathematical primes, whereas this difference is not as big in highly math anxious children and appears to be opposite in DD children. However, since none of these differences reached significance, we cannot confirm an affective priming or a negative affective priming effect in the present study. Nonetheless, differences to Rubinsten and Henik (2005) must also be taken into account. For instance, in their study children were older than the ones included in the present study. As it is assumed that mathematics anxiety increases with age in childhood, the *negative math priming effect* might also be age dependent. Although we included age as a confounding variable in our analyses, age differences are still relevant for consideration of the *negative math priming effect*. In addition, the sample sizes differed as well. Rubinsten and Tannock (2010) included 23 children, whereas we considered data of 172 children. Our large sample size positively influences statistical power and the reliability of our results.

Importantly, the presentation of primes was different between both studies since Rubinsten and Tannock (2010) presented them visually whereas in our study the presentation was aural. This adjustment was made because our children were younger and hence potential difficulties in reading could be excluded through the aural presentation of primes. However, since the above mentioned congruity effects and the Stroop effect were both validated when visual primes were used, the aural presentation in our study might have affected priming effects. With regard to the literature, similar mechanisms have been shown to operate in visual and aural priming, but several differences in the priming effect have also been reported between the two modalities (Holcomb and Neville, 1990). In Holcomb and Neville's study, priming effects were larger in the aural condition but mean reaction times were slower in the aural than in the visual modality. Regarding the different presentation modalities between both studies, the interval between the onset of the prime word and the onset of the target (SOA), as well as the interval between the offset of the word and the onset of the target (ISI) also differed. Whereas in the present study the mean SOA was around 1 s and the ISI ranged from 0 to 567 ms, Rubinsten & Tannock used a shorter SOA of 250 ms and ISI of 0 ms. Klauer (1997) reported in his review that some priming experiments failed to obtain priming effects when using longer SOAs. This has also to be considered in the interpretation of the present findings. However, several studies presenting spoken prime words used comparable timing as in the present study and reported priming effects (Holcomb and Neville (1990) SOA = 1,420–1,850 ms, ISI = 1,150 ms; Voyer and Myles (2017) SOA = 800–1,000 ms, ISI = 50–250 ms; Kim and Sumner (2017) SOA = not indicated, ISI = 100 ms; Bacovcin et al. (2017) SOA = not indicated, ISI = 400–600 ms). Holcomb and Neville (1990) even reported stronger priming effects for auditory (SOA 1,420–1,850 ms) than visual prime words (SOA 1,550 ms), which had even longer SOA. Therefore, the longer SOA in the present study are unlikely to be the reason for absent priming effects, but should be further investigated.

Due to the younger age of the participants, we also simplified the task by excluding multiplications and divisions, which resulted in fewer trials (80 compared to 160). Nevertheless, this should not be associated with insufficient power because in contrast to the lesser number of trials per person, we included many more participants in total.

A discrepancy that might have influenced the priming effects is the choice of language for the primes since Rubinsten and Tannock's study (Rubinsten and Tannock, 2010) was conducted in English and the present one in German (for a discussion of this effect for English and Hebrew see also Rubinsten et al., 2012). Certain characteristics of the languages, such as word length, frequency or part of speech (e.g., verbs, nouns or adjectives), may have influenced the priming effects. Moreover, priming words also differed between the studies. In the present study, the words for each category were carefully selected and evaluated in a pilot study with children to confirm that the chosen German words were affectively loaded either positive, negative, or neutral or clearly related to mathematics for children in the age range of the current study. Hence, language and words might influence the priming effects but as priming words were validated and pretested for valence, the language and selection of words should not be the sole cause of the inconsistent findings between the studies. Nevertheless, this issue needs further investigation.

To the best of our knowledge, apart from Rubinsten and Tannock (2010), the only other study that used an affective priming task and included mathematics-related words was conducted by Rubinsten et al. (2012). In spite of standard affective priming effects being observed by that study, no significant effects were found for mathematics-related primes. Thus, in line with the present finding, no *negative math priming effect* was evident.

In summary, the present study observed no priming effects and particularly not the *negative math priming effect* of concern in children with or without DD. However, we found that control children were significantly slower after the presentation of a mathematics-related prime compared to the positive / negative / neutral primes. In contrast, dyscalculic children were slower after a positive, negative or mathematics-related prime compared to a neutral prime. The present findings indicate that an affective math priming task, which is supposed to test the relation between emotions and mathematical performance in an implicit manner, might not be an ideal way to assess mathematics anxiety in the assessed age group. Rather, it might be more reliable to assess mathematics anxiety with explicit measures such as questionnaires.

A more detailed knowledge of the constructs is critical since “an understanding of the effects of math anxiety is fundamental to understand the human cognitive apparatus in numerical abilities,” as pointed out by Rubinsten and Tannock (2010, p. 10). Future research should also address the direction of causation between mathematical performance, mathematics anxiety and negative emotions. Because of the relatively high occurrence of math anxiety, an improved understanding of how these aspects relate to one another would enable interventions to be applied to improve performance in mathematics by reducing math anxiety and negative attitudes toward mathematics in school or math related situations in daily life.

## Author contributions

KK: Conceptualization of the study idea and design, data collection, data analyses and interpretation and preparation and revision of this manuscript. IZ: Data analyses and interpretation and preparation of this manuscript. JK: Conceptualization of the study idea and design, data collection and revision of this manuscript. AW: Conceptualization of the study design and revision of this this manuscript. NP: Conceptualization of the study design and revision of this this manuscript. GE: Conceptualization of the study design and revision of this this manuscript. MvA: Conceptualization of the study design and revision of this this manuscript. All authors have contributed and have approved this final manuscript.

### Conflict of interest statement

The authors declare that the research was conducted in the absence of any commercial or financial relationships that could be construed as a potential conflict of interest.
