# Supplementary material for: Relation Between Mathematical Performance, Math Anxiety, and Affective Priming in Children With and Without Developmental Dyscalculia
Source: Front Psychol. 2018 Apr 26;9:263. doi: 10.3389/fpsyg.2018.00263 (PMC5932531; doi:10.3389/fpsyg.2018.00263)
Supplement: Supplementary file 1 [file Data_Sheet_1.docx]

Supplementary Material

Relation between mathematical performance and emotions in children with and without developmental dyscalculia

Karin Kucian^*^, Isabelle Zuber, Juliane Kohn, Nadine Poltz, Anne Wyschkon, Günter Esser, Michael von Aster

*** Correspondence:** Karin Kucian: [karin.kucian@kispi.uzh.ch](mailto:karin.kucian@kispi.uzh.ch)

# 1. Affective priming split by arithmetic operations

As illustrated in table S1, in the *control group* response latencies were significantly shorter for positive than for math affective primes when the arithmetic operations were either addition or subtraction. Furthermore, response latencies were significantly shorter for neutral than for mathematics-related affective primes for either addition or subtraction. Response latencies were significantly shorter for neutral than for negative primes when the arithmetic operation was addition and significantly shorter for negative than for mathematics-related primes when the arithmetic operation was subtraction.

In the *DD group*, response latencies were significantly shorter for neutral affective primes than for negative affective primes for addition or subtraction. Moreover, response latencies were significantly shorter for neutral than for positive affective primes for subtraction.

**Table S1*.* Post hoc tests for type of prime (valence) depending on the arithmetic operation.**

|  | | **CC** | **DD** | |
| --- | --- | --- | --- | --- |
| **Operation** | **Valence** | **RT Difference Statistics**  **(ms)** | | **RT Difference Statistics**  **(ms)** |
| **Addition** | **Positive - Negative**  **Positive - Neutral**  **Positive - Math**  **Negative - Neutral**  **Negative - Math**  **Neutral - Math** | -56.2 n.s.  8.5 n.s.  -86.6 *t*(93) = -3.17*, p* <.05  59.8 *t*(92) = 2.35*, p* <.05  -42.7 n.s.  -98.6 *t*(94) = -3.57*, p* <.001 | | -58.8 n.s.  59.2 n.s.  -11.5 n.s.  114.1 *t*(74) = 2.39*, p* <.05  47.2 n.s.  -66.9 n.s. |
| **Subtraction** | **Positive - Negative**  **Positive - Neutral**  **Positive - Math**  **Negative - Neutral**  **Negative - Math**  **Neutral - Math** | -4.1 n.s.  57.5 n.s.  -77.7 *t*(93) = -2.14*, p* <.05  61.5 n.s.  -73.6 *t*(93) = -2.20*, p* <.05  -135. *t*(93) = -3.97*, p* < .001 | | -26.9 n.s.  118.2 *t*(72) = 2.27*, p* <.05  -.2 n.s.  147.0 *t*(70) = 2.96*, p* <.05  30.7 n.s.  -86.1 n.s. |

# Differences between arithmetic operations split by primes

As shown in table S2, for each group the reaction times between addition and subtraction were analysed separately for each type of prime. In the *control group*, significantly shorter response latencies were found for addition than for subtraction across all types of prime.

In the *DD group* the response latencies between operations did not differ depending on the prime.

**Table S2. Post hoc tests for the arithmetic operations (valence) depending on the type of prime.**

|  | | **CC** | **DD** |
| --- | --- | --- | --- |
| **Prime** | **Valence** | **RT Difference Statistics**  **(ms)** | **RT Difference Statistics**  **(ms)** |
| **Positive** | **Addition-Subtraction** | -132.9 *t*(92) = -3.59*, p* <.001 | -70.5 n.s. |
| **Negative** | **Addition-Subtraction** | -87.7 *t*(92) = -3.22*, p* <.05 | -54.4 n.s. |
| **Neutral** | **Addition-Subtraction** | -77.9 *t*(93) = -2.27*, p* <.05 | -23.5 n.s. |
| **Math** | **Addition-Subtraction** | -131.2 *t*(94) = -3.97*, p* < .001 | -62.9 n.s. |

# Data analyses for accuracy levels above chance

Only children that performed above chance level (mean accuracy ≥ 50%) in the affective priming task were included in the following analyses. Table S3 summarizes the demographic and behavioural data of this sub cohort. Repeated measures ANCOVA with type of prime (positive / negative / neutral / mathematics-related) and arithmetic operation (addition / subtraction) as within-subject factors, group (CC / DD) as between-subject factor and age as a covariate showed no significant effects or interactions. However, the interaction between type of prime and group missed significance only narrowly (*F*(3,142) = 2.654, *p* = .051, *η^2^* = .053).

**Table S3. Demographic and behavioural data of the dyscalculic children (DD) and control children (CC), accuracy ≥ 50%.**

|  | **Total** | **DD** | **CC** | **Statistics** |
| --- | --- | --- | --- | --- |
| **Subjects (N)** | 148 | 59 | 89 |  |
| **Age (years) *M* (*SD*)** | 8.64 (0.97) | 9.2 (1.05) | 8.27 (0.72) | *U* = 1215.5, *z* = - 5.53, *p* <.001 |
| **Gender (male / female)** | 49 / 99 | 19 / 40 | 30 / 59 | *χ*^2^(1, *N*=148) = 0.04, n.s. |
| **Intelligence (IQ) ^a^ *M* (*SD*)** | 101.59 (7.60) | 97.22 (5.81) | 104.49 (7.27) | *U* = 1157.5, *z* = - 5.75, *p* <.001 |
| **Mathematical performance (T) ^b^ *M* (*SD*)** | 44.75 (9.27) | 35.41 (3.76) | 50.94 (6.08) | *U* < 0.001, *z* = -10.28, *p* <.001 |
| **Math anxiety (intensity) ^c^ *M* (*SD*)** | 2.99 (2.49) | 4.03 (2.49) | 2.29 (2.24) | *U* = 1540, *z* = -4.26, *p* <.001 |
| **Arithmetic fluency (*T*) ^d^ *M* (*SD*)** | 42.73 (10.05) | 33.93 (5.48) | 48.58 (7.91) | *t*(146) = 13.3, *p* <.001 |
| **Addition (% correct) ^e^ *M* (*SD*)** | 85.88 (17.14) | 75.18 (21.23) | 93.05 (7.93) | *U* = 892, *z* = -6.08, *p* <.001 |
| **Subtraction (% correct) ^e^ *M* (*SD*)** | 75.07 (21.29) | 59.45 (21.75) | 85.55 (13.01) | *U* = 697, *z* = -6.87, *p* <.001 |
| **Number line (% deviation) ^e^ *M* (*SD*)** | 4.49 (2.46) | 5.42 (2.86) | 3.86 (1.94) | *U* = 1516, *z* = -3.26, *p* <.001 |
| **Number line (*T*) ^f^ *M* (*SD*)** | 57.22 (14.39) | 49.29 (13.51) | 62.48 (12.46) | *U* = 1113, *z* = -5.93, *p* <.001 |
| **Working memory (items) ^g^ *M* (*SD*)** | 4.17 (1.6) | 4 (1.52) | 4.28 (1.65) | *U* = 2419.5, *z* = -0.43, n.s. |

^a^ Mean IQ based on 4 subtests (verbal IQ and matrices test of BUEGA, block design and similarities subtest of WISC-IV (N=131)), or based on 6 subtests of the WISC-IV (block design, similarities, digit span, picture concepts, vocabulary, arithmetic (N=17)).

^b^ Mean mathematical performance based on 4 subtests (addition and subtraction of HRT, Zahlenstrahl II of ZAREKI-R, Rechentest of BUEGA) (N=131), or based on addition and subtraction of HRT and ZAREKI-R (N=17) in T-values.

^c^ Mean intensity of math anxiety assessed by the math anxiety interview (MAI), whereas 0 = no math anxiety, 10 = very high math anxiety.

^d^ Mean of addition and subtraction of the HRT in *T*-values.

^e^ Based on number line task. The percentage of correctly solved addition or subtraction problems are listed. Moreover, the percentage of the deviation between the exact location on the number line and the marked location of the child is indicated.

^f^ Based on subtests number line I and II of ZAREKI-R in *T*-values.

^g^ Based on maximum number of correctly recalled items of the Corsi-Suppression test.

# Gender differences

Tables S4 and S5 summarize demographic and behavioural data for female and male control and DD children, respectively.

**Table S4. Demographic and behavioural data of the control children (CC), separated by gender.**

|  | **Total CC** | **CC females** | **CC males** | **Statistics** |
| --- | --- | --- | --- | --- |
| **Subjects (N)** | 96 | 66 | 30 |  |
| **Age (years) M (SD)** | 8.23 (0.71) | 8.23 (0.75) | 8.23 (0.63) | *U* = 960.5, *z* = - 0.233, n.s. |
| **Intelligence (IQ) ^a^ *M* (*SD*)** | 104.65 (7.16) | 105.18 (6.94) | 103.49 (7.6) | *t*(94) = 1.08, n.s. |
| **Mathematical performance (*T*) ^b^ *M* (*SD*)** | 50.83 (6.00) | 50.22 (5.33) | 52.17 (7.19) | *t*(94) = -1.33, n.s. |
| **Math anxiety (intensity) ^c^ *M* (*SD*)** | 2.35 (2.24) | 2.33 (1.97) | 2.38 (2.78) | *U* = 870.5, *z* = -.95, n.s. |
| **Arithmetic fluency (*T*) ^d^ *M* (*SD*)** | 48.13 (7.84) | 46.91 (7.36) | 50.82 (8.33) | *U* = 730, *z* = -2.06, *p* <.05 |
| **Addition (% correct) ^e^ *M* (*SD*)** | 92.30 (8.53) | 91.94 (9.42) | 93.15 (6.07) | *U* = 828.5, *z* = -0.08, n.s. |
| **Subtraction (% correct) ^e^ *M* (*SD*)** | 85.11 (13.08) | 83.63 (13.97) | 88.52 (10.17) | *U* = 677, *z* = -1.44, n.s. |
| **Number line (% deviation) ^e^ *M* (*SD*)** | 3.91 (1.92) | 3.94 (1.75) | 3.83 (2.3) | *U* = 764.5, *z* = -0.65, n.s. |
| **Number line (*T*) ^f^ *M* (*SD*)** | 62.87 (12.20) | 63.35 (12.13) | 61.83 (12.49) | *U* = 955, *z* = -0.28, n.s. |
| **Working memory (items) ^g^ *M* (*SD*)** | 4.28 (1.68) | 4.25 (1.70) | 4.33 (1.67) | *U* = 878, *z* = -0.69, n.s. |

^a^ Mean IQ based on 4 subtests (verbal IQ and matrices test of BUEGA, block design and similarities subtest of WISC-IV (N=86)), or based on 6 subtests of the WISC-IV (block design, similarities, digit span, picture concepts, vocabulary, arithmetic (N=10)).

^b^ Mean mathematical performance based on 4 subtests (addition and subtraction of HRT, Zahlenstrahl II of ZAREKI-R, Rechentest of BUEGA), or based on addition and subtraction of HRT and ZAREKI-R in *T*-values.

^c^ Mean intensity of math anxiety assessed by the math anxiety interview (MAI), whereas 0 = no math anxiety, 10 = very high math anxiety.

^d^ Mean of addition and subtraction of the HRT in *T*-values.

^e^ Based on number line task. The percentage of correctly solved addition or subtraction problems are listed. Moreover, the percentage of the deviation between the exact location on the number line and the marked location of the child is indicated.

^f^ Based on subtests number line I and II of ZAREKI-R in *T*-values.

^g^ Based on maximum number of correctly recalled items of the CORSI-Suppression test.

**Table S5. Demographic and behavioural data of the dyscalculic children (DD), separated by gender.**

|  | **Total DD** | **DD females** | **DD males** | **Statistics** |
| --- | --- | --- | --- | --- |
| **Subjects (N)** | 76 | 54 | 22 |  |
| **Age (years) *M* (*SD*)** | 9.04 (1.03) | 9.02 (1.03) | 9.07 (1.06) | *t*(74) = -0.18, n.s. |
| **Intelligence (IQ) ^a^ *M* (*SD*)** | 96.53 (5.73) | 96.92 (5.56) | 95.59 (6.17) | *t*(74) = 0.92, n.s. |
| **Mathematical performance (T) ^b^ *M* (*SD*)** | 34.99 (3.65) | 35.32 (3.44) | 34.45 (4.17) | *U* = 539.5 *z* = -0.62, n.s. |
| **Math anxiety (intensity) ^c^ *M* (*SD*)** | 4.37 (2.41) | 4.49 (2.27) | 4.08 (2.75) | *U* = 550.5, *z* = -0.50, n.s. |
| **Arithmetic fluency (*T*) ^d^  *M* (*SD*)** | 33.35 (5.71) | 34.25 (5.48) | 31.14 (5.79) | *t*(74) = 2.209, *p <.*05 |
| **Addition (% correct) ^e^ *M* (*SD*)** | 68.31 (23.65) | 69.71 (23.02) | 64.74 (25.54) | *U* = 440, *z* = -0.70, n.s. |
| **Subtraction (% correct) ^e^ *M* (*SD*)** | 54.51 (22.76) | 57.31 (21.95) | 46.84 (23.76) | *t*(69) = 1.74, n.s. |
| **Number line (% deviation) ^e^ *M* (*SD*)** | 5.77 (3.03) | 5.89 (3.12) | 5.44 (2.83) | *U* = 470, *z* = -0.31, n.s. |
| **Number line (T) ^f^ *M* (*SD*)** | 47.19 (12.71) | 46.81 (12.66) | 48.12 (13.08) | *U* = 553, *z* = -0.47, n.s. |
| **Working memory (items) ^g^ *M* (*SD*)** | 3.77 (1.58) | 3.98 (1.60) | 3.24 (1.45) | *U* = 365, *z* = -2.48, *p* <.05 |

^a^ Mean IQ based on 4 subtests (verbal IQ and matrices test of BUEGA, block design and similarities subtest of WISC-IV (N=67)), or based on 6 subtests of the WISC-IV (block design, similarities, digit span, picture concepts, vocabulary, arithmetic (N=9)).

^b^ Mean mathematical performance based on 4 subtests (addition and subtraction of HRT, Zahlenstrahl II of ZAREKI-R, Rechentest of BUEGA), or based on addition and subtraction of HRT and ZAREKI-R in *T*-values.

^c^ Mean intensity of math anxiety assessed by the math anxiety interview (MAI), whereas 0 = no math anxiety, 10 = very high math anxiety.

^d^ Mean of addition and subtraction of the HRT in *T*-values.

^e^ Based on number line task. The percentage of correctly solved addition or subtraction problems are listed. Moreover, the percentage of the deviation between the exact location on the number line and the marked location of the child is indicated.

^f^ Based on subtests number line I and II of ZAREKI-R in *T*-values.

^g^ Based on maximum number of correctly recalled items of the CORSI-Suppression test.

# Mathematics anxiety

**Table S6. Demographic and behavioural data, separated in low vs. high mathematics** **anxiety children (MAI).**

|  | **Low & High MAI group** | **Low MAI group** | **High MAI group** | **Statistics** |
| --- | --- | --- | --- | --- |
| **Subjects (N)** | 100 | 46 | 54 |  |
| **Age (years) *M* (*SD*)** | 8.57 (0.97) | 8.5 (1.10) | 8.63 (0.85) | *U* = 1018.5, *z* = -1.55, n.s. |
| **Gender (male / female)** | 35 / 65 | 18 / 28 | 17 / 37 | χ*^2^*(1*, N=*100) = 0.64, n.s. |
| **Group (CC / DD)** | 49 / 51 | 34 / 12 | 15 / 39 | χ*^2^*(1*, N=*100) = 21.16, *p* <.001 |
| **Intelligence (IQ) ^a^ *M* (*SD*)** | 100.6 (7.73) | 103.53 (7.61) | 98.1 (6.98) | *U* = 738, *z* = -5.92, p <.001 |
| **Mathematical performance (*T*) ^b^ *M* (*SD*)** | 43.25 (10.30) | 49.82 (9.51) | 37.65 (7.21) | *U* = 386, *z* = -5.82, *p* <.001 |
| **Math anxiety (intensity) ^c^  *M* (*SD*)** | 3.55 (3.15) | 0.33 (0.34) | 6.29 (1.32) | *U* < 0.001, *z* = -8.63, *p* <.001 |
| **Arithmetic fluency (*T*) ^d^  *M* (*SD*)** | 41.34 (11.22) | 48.32 (10.56) | 35.39 (7.89) | *t*(82) = 6.99, *p* <.001 |
| **Addition (% correct) ^e^  *M* (*SD*)** | 77.93 (23.54) | 92.62 (6.74) | 65.6 (25.51) | *U* = 331.5, *z* = -5.68, *p* <.001 |
| **Subtraction (% correct) ^e^  *M* (*SD*)** | 69.08 (25.45) | 86.19 (13.96) | 54.7 (24.04) | *U* = 257.5, *z* = -6.24, *p* <.001 |
| **Number line (% deviation) ^e^ *M* (*SD*)** | 5.1 (2.94) | 4.08 (1.97) | 5.96 (3.31) | *U* = 688, *z* = -2.84, *p* = .005 |
| **Number line (*T*) ^f^ *M* (*SD*)** | 54.45 (14.29) | 61.19 (12.23) | 48.7 (13.46) | *U* = 573, *z* = -4.63, *p* < .001 |
| **Working memory (items) ^g^ *M* (*SD*)** | 3.87 (1.72) | 4.47 (1.59) | 3.37 (1.67) | *U* = 778.5, *z* = -3.18, *p <*.001 |

^a^ Mean IQ based on 4 subtests (verbal IQ and matrices test of BUEGA, block design and similarities subtest of WISC-IV (N=89)), or based on 6 subtests of the WISC-IV (block design, similarities, digit span, picture concepts, vocabulary, arithmetic (N=11)).

^b^ Mean mathematical performance based on 4 subtests (addition and subtraction of HRT, Zahlenstrahl II of ZAREKI-R, Rechentest of BUEGA), or based on addition and subtraction of HRT and ZAREKI-R in *T*-values.

^c^ Mean intensity of math anxiety assessed by the math anxiety interview (MAI), whereas 0 = no math anxiety, 10 = very high math anxiety.

^d^ Mean of addition and subtraction of the HRT in *T*-values.

^e^ Based on number line task. The percentage of correctly solved addition or subtraction problems are listed. Moreover, the percentage of the deviation between the exact location on the number line and the marked location of the child is indicated.

^f^ Based on subtests number line I and II of ZAREKI-R in *T*-values.

^g^ Based on maximum number of correctly recalled items of the Corsi-Suppression test.

1. **Priming words**

**Table S7*.* Priming word list**

| **Valence** | ***Negative*** | ***Positive*** | ***Neutral*** | ***Mathematics*** |
| --- | --- | --- | --- | --- |
| **Words** | Tod  Krieg  Sterben  Gefängnis Unfall  streiten  verletzen  schlagen  krank  dumm | Geschenk  Spass  Party  freuen  Sonne  Ferien  lachen  spielen  lustig  gewinnen | Deckel  Dose  stehen  Strasse  Wand  Kiste  Schrank  gehen  Boden  Holz | minus  Mathe  zählen  lösen  plus  Ergebnis  Zahl  Rechnen  Geteilt  Viereck |
| **Letters** | 64 | 61 | 52 | 58 |
| **Syllables** | 21 | 21 | 17 | 19 |
| **Noun / Verb / Adjective** | 4 / 4 / 2 | 5 / 4 / 1 | 8 / 2 | 4 / 3 / 3 |
| **Mean rating, *SD*** | 0.59 (0.34) | 3.71 (0.36) | 2.36 (0.43) | 2.63 (0.62) |
